# Monophasic Synovial Sarcoma of Prostatic Fascia: Case Report and Literature Review

**DOI:** 10.1155/2015/419180

**Published:** 2015-05-14

**Authors:** Lucio Olivetti, Luigi Benecchi, Serena Corti, Carlo Del Boca, Matteo Ferrari, Pietro Sergio, Luisa Bercich, Giulia Tanzi

**Affiliations:** ^1^Department of Radiology, Cremona Hospital, Viale Concordia 1, 26100 Cremona, Italy; ^2^Department of Urology, Cremona Hospital, Viale Concordia 1, 26100 Cremona, Italy; ^3^Department of Radiology, University of Brescia, “Spedali Civili” Hospital, Piazzale Spedali Civili 1, 25100 Brescia, Italy; ^4^Department of Anatomic Pathology, “Spedali Civili” Hospital, Piazzale Spedali Civili 1, 25100 Brescia, Italy; ^5^Department of Anatomic Pathology, Cremona Hospital, Viale Concordia 1, 26100 Cremona, Italy

## Abstract

Synovial sarcoma (SS) primarily occurs in the para-articular soft tissue of the lower extremities in young adults and it is extremely rare in the prostatic region. We report a case of a 46-year-old man who presented with urinary retention. Pelvic ultrasound (US) examination, computed tomography (CT), and magnetic resonance imaging (MRI) demonstrated an 8.5 cm mass that appeared to originate in the prostatic fascia of the right lobe. Preoperative prostatic ultrasound transrectal needle biopsy revealed mesenchymal neoplastic tissue. Patient underwent surgery. The final pathologic findings were consistent with the diagnosis of monophasic synovial sarcoma.

## 1. Introduction

Synovial sarcoma (SS) is a soft tissue sarcoma of uncertain histogenesis, chiefly occurring in young adults, primarily in the para-articular region of the extremities. Other unusual sites include the middle ear, orofacial or oropharyngeal region, esophagus, larynx, lung, pleura, heart, blood vessels, abdominal wall, gastrointestinal tract, and retroperitoneum; involvement of the lower genitourinary tract is exceedingly rare. Although a variety of sarcomas, including rhabdomyosarcoma, fibrosarcoma, leiomyosarcoma, and stromal sarcoma, have been described in the prostate, SS is a rare occurrence, with only seven previously reported cases [1–6]. The purpose of our paper is to add an additional case and to review the literature.

## 2. Case Report

**Figure 1:**
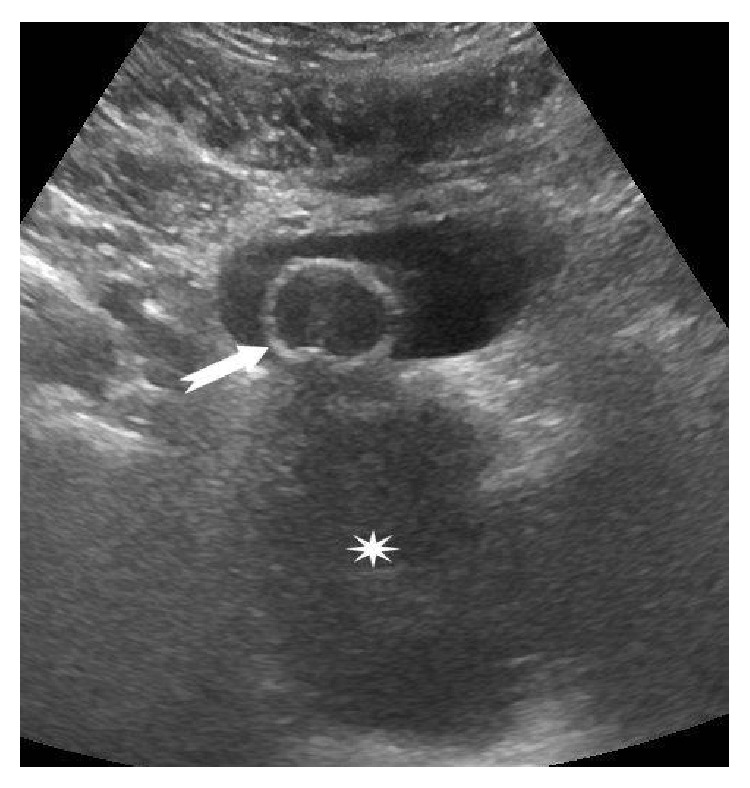
Pelvic ultrasound. A large hypoechoic mass (asterisk) is seen in the prostatic loggia. The arrow indicates the urinary catheter.

A 46-year-old man presented with urinary retention. On digital rectal examination a large prostatic mass was palpable. His serum prostate-specific antigen (PSA) level was 1.03 ng/mL (normal 0–4). Pelvic ultrasound (US) showed an 8 × 7.5 × 8.5 cm lesion localized in the prostatic region and behind the bladder with heterogeneous mixed echogenicity (Figure 1).

**Figure 2:**
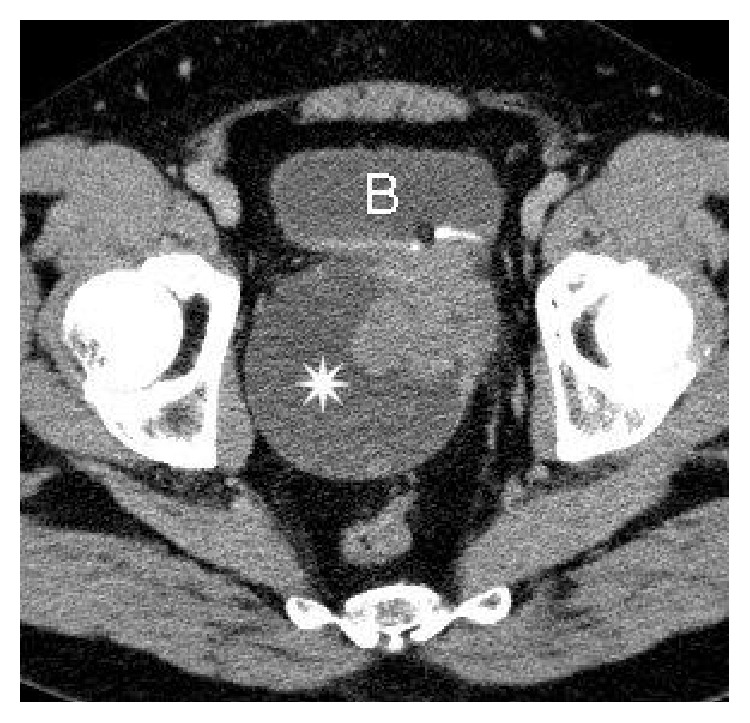
CT of the pelvis, enhanced axial image. The asterisk is on the cystic component of the mass. The arrow shows the solid part of the lesion. B = bladder.

Computed tomography (CT) demonstrated a well-marginated soft tissue tumor that appeared to originate in the prostate and extend to the retrovesical soft tissue, with fluid cystic and solid structure (Figure 2).

No metastasis is evident. Magnetic resonance imaging (MRI) confirmed the origin of the mass in the prostatic fascia of the right prostatic lobe, its prevalent cystic myxoid component with septa and irregular eccentric solid tissue homogeneously enhanced after gadolinium. Right seminal vesicle was compressed.

**Figure 3:**
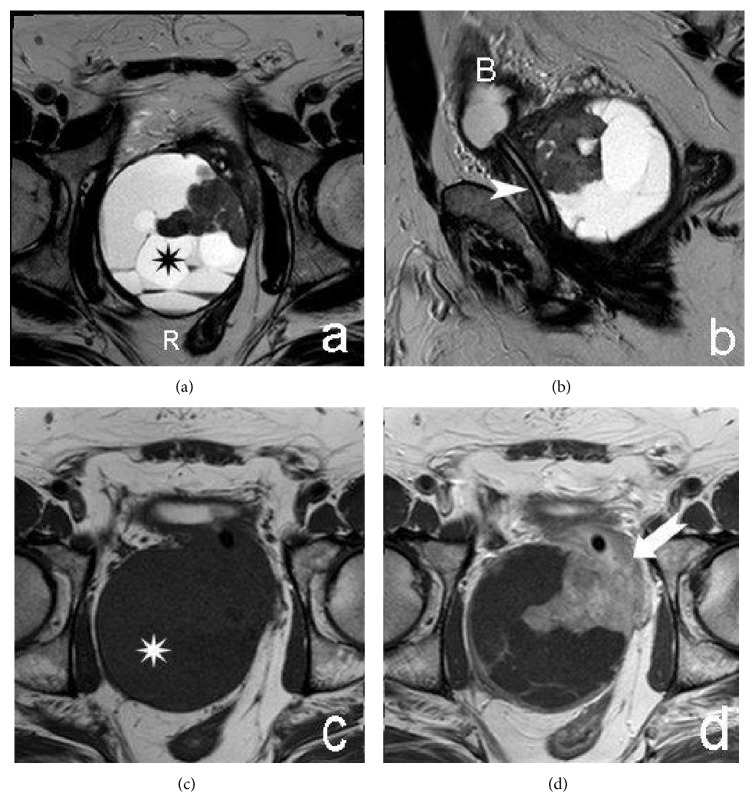
MRI. Axial (a) and sagittal (b) T2-weighted images. Axial precontrast (c) and postcontrast (d) T1-weighted images. The asterisk is on the cystic component of the prostatic lesion; septa are evident. The arrow shows the solid component that improves after Gadolinium injection. Note the presence of a urinary catheter (arrowhead). B = bladder; R = rectum.

Posteriorly the lesion was in close contact with the rectum, laterally with the right obturator internus muscle and inferiorly with the levator ani muscle (Figure 3).

Preoperative prostatic ultrasound transrectal needle biopsy revealed mesenchymal neoplastic tissue. Patient refused to receive radical prostatectomy.

The patient underwent debulking surgical removal. The extension of the mass has prevented its complete removal.

**Figure 4:**
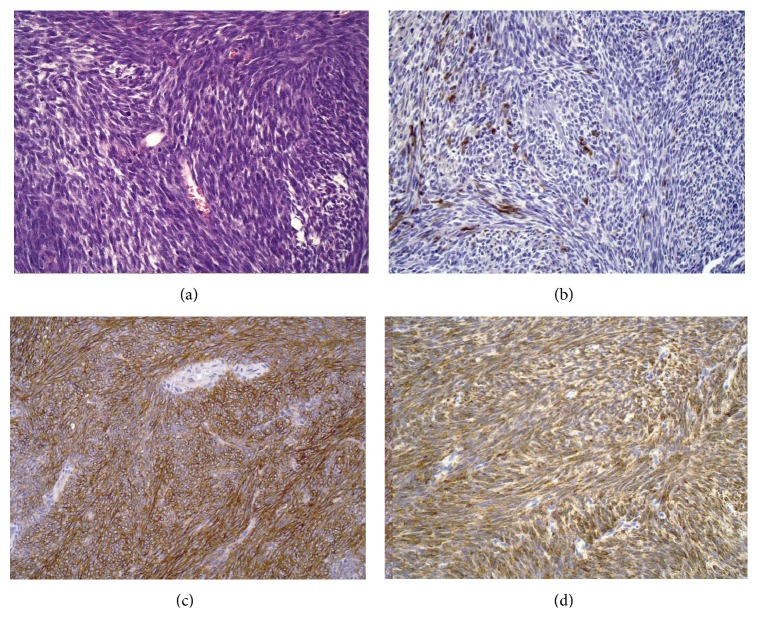
The spindle cells are small, uniform, and closely packed and have a high nuclear-cytoplasmic ratio (a). Immunohistochemical staining showing scattered spindle cells positive for CK AE1/AE3 (b). Diffuse expression of BCL-2 (c) and CD99 (d) in spindle cells.

Routine hematoxylin eosin stained microscopical sections showed a spindle cell neoplasm made up of small, uniform spindle cells with a high nuclear-cytoplasmic ratio, and finely stippled chromatin. The neoplastic cells were closely packed, growing in short interlacing and intersecting fascicles around a branching, “hemangiopericytoma-like vasculature” (Figure 4(a)). The mitotic rate was 1-2 mitoses/10 HPFs. The tumor showed no glandular differentiation. Necrosis was not seen. Immunohistochemical analysis showed pankeratin expression confined to scattered, individual spindle cells (Figure 4(b)), and diffuse expression of CD56, CD99 (Figure 4(c)), and BCL-2 (Figure 4(d)). S100 protein, muscle actin, desmin, and CD34 were entirely negative.

**Figure 5:**
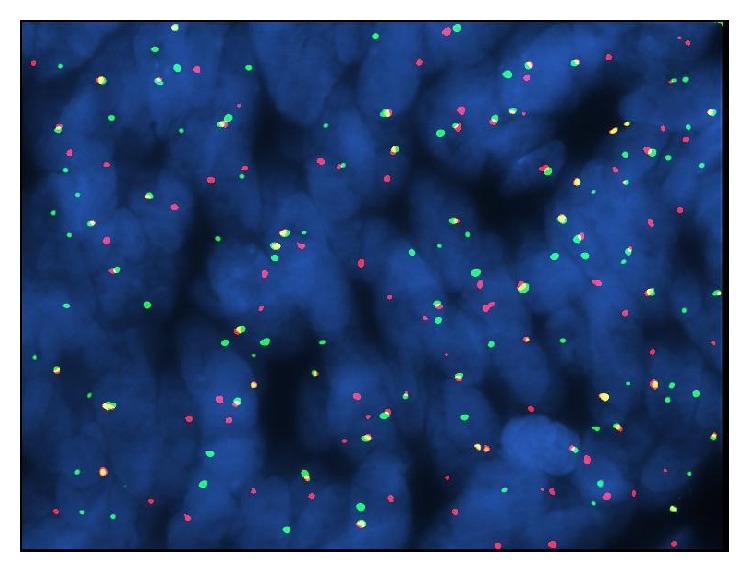
Photomicrograph of FISH results showing splitting of the fluorescent signal revealing SYT rearrangement.

The fluorescence in situ hybridization (FISH) test for t(X;18)(p11q11) was positive showing splitting of the fluorescent signal consistent with a rearrangement involving SYT (Figure 5). The pathologic findings were consistent with the diagnosis of monophasic synovial sarcoma, with positive surgical margins.

**Figure 6:**
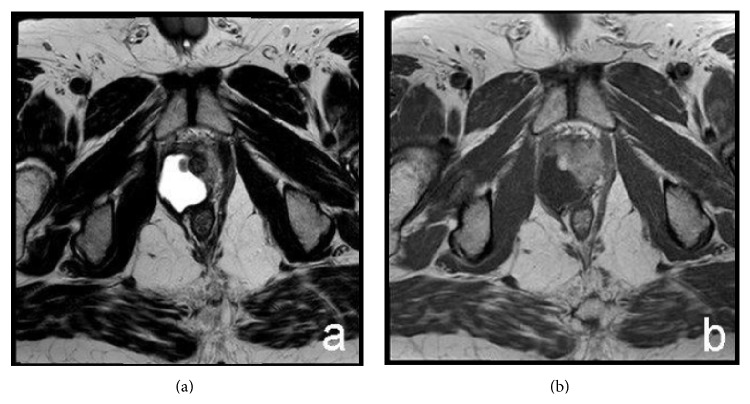
MRI. Postoperative images. Axial T2-weighted (a) and T1-weighted after contrast administration (b) images. Persistence of synovial sarcoma in the prostatic loggia.

Postoperative chemotherapy with epirubicin (25 mg/m^2^) and ifosfamide (2500 mg/m^2^) was offered at days 1, 2, and 3 of fortnightly cycles. At 3 months of follow-up, after 6 cycles of chemotherapy, pelvic RMI showed persistence of the neoplasm, with cystic and solid component, confined in the prostatic loggia and with invasion of the right prostatic lobe (Figure 6). Prostatic ultrasound transrectal needle biopsy confirmed the presence of monophasic synovial sarcoma. Positron emission tomography (PET) was negative for distant metastases.

## 3. Discussion

Synovial sarcoma is a clinically and histomorphologically well-defined soft tissue tumor that has been shown to account for 8% of all soft-tissue sarcoma [2]. Despite its name, the lesion does not commonly arise in an intra-articular location but usually occurs near joints. About 80–95% of SS affect the extremities of adolescents and young adults (15–40 years old) with extensive metastatic potential, especially to the lung and lymph node. [7]. SS also arises in areas with no obvious synovial or periarticular structures; with the aid of immunohistochemistry and, more recently, demonstration of the specific t(X;18) chromosomal translocation or resulting SYT/SSX fusion gene transcripts, this tumor is described in almost all parts of the body [8]. Synovial sarcoma arising primarily from the prostate is a rare occurrence, with only seven previously reported cases [1–6], the majority of which are monophasic. Histologic subtypes include biphasic (20–30%), monophasic (the most common, 50–60%), and poorly differentiated SS (15–25%). Biphasic SS in varying proportions has both a mesenchymal spindle cell component and an obvious epithelial component; the monophasic subtype consists solely of mesenchymal spindle cells. [9]. Gross pathologic appearance of SS is nonspecific but frequently is multilobulated with areas of necrosis, hemorrhage, cyst formation, and focal tumor calcification [2]. In our case, spindle cell occurred alone, with ovoid nuclei, fascicular growth pattern, and mild mitotic activity.

The cytogenetic analysis with the break apart FISH technique confirmed the SYT locus translocation, an aberration that is specific for synovial sarcoma [1, 2, 6].

In our patient the PSA was normal as in the seven previously described cases. It has been reported that the PSA level may not rise in patients with prostatic sarcoma due to the nonepithelial origin of the neoplasm [10].

In this case cross sectional imaging was helpful for staging tumor extent and planning surgical resection. The CT findings were that of a well-marginated, heterogeneous soft-tissue mass where the low-attenuation area was the predominant feature, an appearance that simulated a cystic mass. No calcifications were present. After administration of intravenous contrast material heterogeneous enhancement was observed, a feature that was quite helpful for distinguishing SS from a simple cystic lesion.

MRI assessed the extent and intrinsic characteristics of SS in our patient obviously better than CT. MRI is the modality of choice for the diagnosis and initial staging of SS because of the information provided by intrinsic signal characteristics and superior soft-tissue contrast. On T1-weighted MR images, the lesion appeared as a homogeneous rounded soft tissue mass with signal intensity similar to that of muscle. On T2-weighted images the lesion showed a prominent heterogeneity and predominant hyperintensity due to the cystic component.

Fisher reported that myxoid change was seen in 55% of 11 intra-abdominal SS and in one case was prominent and five (45%) of the tumors of his study were markedly cystic [8].

The signal heterogeneity was described by Jones et al. [11] in their series of 34 patients with soft tissue SS: thirty-five percent of the lesions had areas that were hyper-, iso-, and hypointense relative to fat on T2-weighted images, constituting a triple signal intensity that on T2-weighted MR images is due to the result of the mixture of solid cellular elements (intermediate signal intensity), hemorrhage, or necrosis (high signal intensity), and calcified or fibrotic collagenized regions (low signal intensity). However, the triple sign is also seen in other soft-tissue neoplasms [12].

In a study of 18 SS Liang et al. reported that all cases demonstrated areas of hyperintensity, isointensity, and hypointensity relative to the muscle. Hyperintensive signals on T1-weighted and T2-weighted were observed in necrotic and cystic areas. Nine cases demonstrated a hypointensive T1 signal, suggesting hemorrhage. Three cases with hypointensive T1 signals were considered to possess fibrous tissue. In total, 12 lesions exhibited a hypointensive T2 signal with internal septations. Contrast-enhanced images revealed heterogeneous lesions, with no enhancement in the areas containing cysts, necrosis, or septation [13].

The survival of patients with primary SS of prostatic fascia is poor, within a few months after diagnosis and aggressive resection surgery that remains the current treatment of choice for locally confined lesion. It is uncertain whether chemotherapy has a substantial effect on long-term survival. Documentation of further cases is needed to establish appropriate therapy.

## References

[1] Iwasaki H., Ishiguro M., Ohjimi Y. (1999). Synovial sarcoma of the prostate with t(X;18)(p11.2;q11.2). *American Journal of Surgical Pathology*.

[2] Shirakawa T., Fujisawa M., Gotoh A., Okada H., Arakawa S., Kamidono S. (2003). Complete resection of synovial sarcoma of prostatic fascia. *Urology*.

[3] Williams D. H., Hua V. N., Chowdhry A. A. (2004). Synovial sarcoma of the prostate. *The Journal of Urology*.

[4] Pan C.-C., Chang Y.-H. (2006). Primary synovial sarcoma of the prostate. *Histopathology*.

[5] Jun L., Ke S., Zhaoming W., Linjie X., Xinru Y. (2008). Primary synovial sarcoma of the prostate: report of 2 cases and literature review. *International Journal of Surgical Pathology*.

[6] Zhang Q., Wang H., Ren L., Qi X., Liu F., Zhang D. (2014). Primary synovial sarcoma of the prostate metastatic to the liver and lung: a case report. *World Journal of Surgical Oncology*.

[7] Murphey M. D., Gibson M. S., Jennings B. T., Crespo-Rodríguez A. M., Fanburg-Smith J., Gajewski D. A. (2006). From the archives of the AFIP: imaging of synovial sarcoma with radiologic-pathologic correlation. *Radiographics*.

[8] Fisher C. (1998). Synovial sarcoma. *Annals of Diagnostic Pathology*.

[9] Fisher C., Folpe A. L., Hashimoto H., Weiss S. W. (2004). Intra-abdominal synovial sarcoma: a clinicopathological study. *Histopathology*.

[10] Sexton W. J., Lance R. E., Reyes A. O., Pisters P. W. T., Tu S.-M., Pisters L. L. (2001). Adult prostate sarcoma: the M. D. Anderson cancer center experience. *Journal of Urology*.

[11] Jones B. C., Sundaram M., Kransdorf M. J. (1993). Synovial sarcoma: MR imaging findings in 34 patients. *American Journal of Roentgenology*.

[12] Bakri A., Shinagare A. B., Krajewski K. M. (2012). Synovial sarcoma: imaging features of common and uncommon primary sites, metastatic patterns, and treatment response. *American Journal of Roentgenology*.

[13] Liang C., Mao H., Tan J. (2015). Synovial sarcoma: magnetic resonance and computed tomography imaging features and differential diagnostic considerations. *Oncology Letters*.

